# Ligand bias in receptor tyrosine kinase signaling

**DOI:** 10.1074/jbc.REV120.015190

**Published:** 2021-01-13

**Authors:** Kelly Karl, Michael D. Paul, Elena B. Pasquale, Kalina Hristova

**Affiliations:** 1 Department of Materials Science and Engineering, Institute for NanoBioTechnology, and Program in Molecular Biophysics, Johns Hopkins University, Baltimore, Maryland, USA; 2 Sanford Burnham Prebys Medical Discovery Institute, La Jolla, California, USA

**Keywords:** Receptor tyrosine kinase (RTK), receptor, ligand bias, cell signaling, bias plot, receptor tyrosine kinase, phosphotyrosine signaling, signaling, dimerization, mathematical modeling, protein conformation, bias coefficient, dimer stability, ligand functional selectivity, thermodynamics

## Abstract

Ligand bias is the ability of ligands to differentially activate certain receptor signaling responses compared with others. It reflects differences in the responses of a receptor to specific ligands and has implications for the development of highly specific therapeutics. Whereas ligand bias has been studied primarily for G protein–coupled receptors (GPCRs), there are also reports of ligand bias for receptor tyrosine kinases (RTKs). However, the understanding of RTK ligand bias is lagging behind the knowledge of GPCR ligand bias. In this review, we highlight how protocols that were developed to study GPCR signaling can be used to identify and quantify RTK ligand bias. We also introduce an operational model that can provide insights into the biophysical basis of RTK activation and ligand bias. Finally, we discuss possible mechanisms underpinning RTK ligand bias. Thus, this review serves as a primer for researchers interested in investigating ligand bias in RTK signaling.

“Ligand bias” (also known as “biased agonism” or “ligand functional selectivity”) is the ability of ligands to differentially activate a subset of receptor signaling pathways (1). Research focused on G protein–coupled receptors (GPCRs) has revealed that these seven-transmembrane-helix receptors engage in biased signaling in natural, physiologic systems (2). These discoveries have transformed our understanding of GPCR signaling and have empowered the search for synthetic biased ligands that selectively target therapeutically relevant signaling pathways (2, 3, 4, 5). These new ligands can modify specific signaling responses, leading to different functional and physiological consequences as compared with natural ligands (6).

Receptor tyrosine kinases (RTKs) may also engage in biased signaling (7, 8, 9). The 58 human RTKs represent the second largest family of transmembrane receptors, after the GPCRs. RTKs control cell growth, differentiation, motility, and metabolism by transducing biochemical signals induced upon their dimerization or oligomerization in the plasma membrane (10, 11, 12). Their N-terminal extracellular regions are composed of characteristic arrays of structural domains and bind activating ligands (13, 14) (Fig. 1). RTKs also contain a single transmembrane helix and an intracellular region including the kinase domain. The cross-phosphorylation mediated by the kinase domains in the ligand-bound RTK dimers and oligomers stimulates kinase activity. This leads to further autophosphorylation on multiple tyrosines in the intracellular region as well as phosphorylation of cytoplasmic substrates, thus activating signaling cascades that ultimately control cell behavior (14, 15, 16). Given that RTKs control so many critical aspects of cell communication and function, it is not surprising that they have been implicated in many developmental disorders and cancers (10, 16, 17, 18, 19, 20).

**Figure 1:**
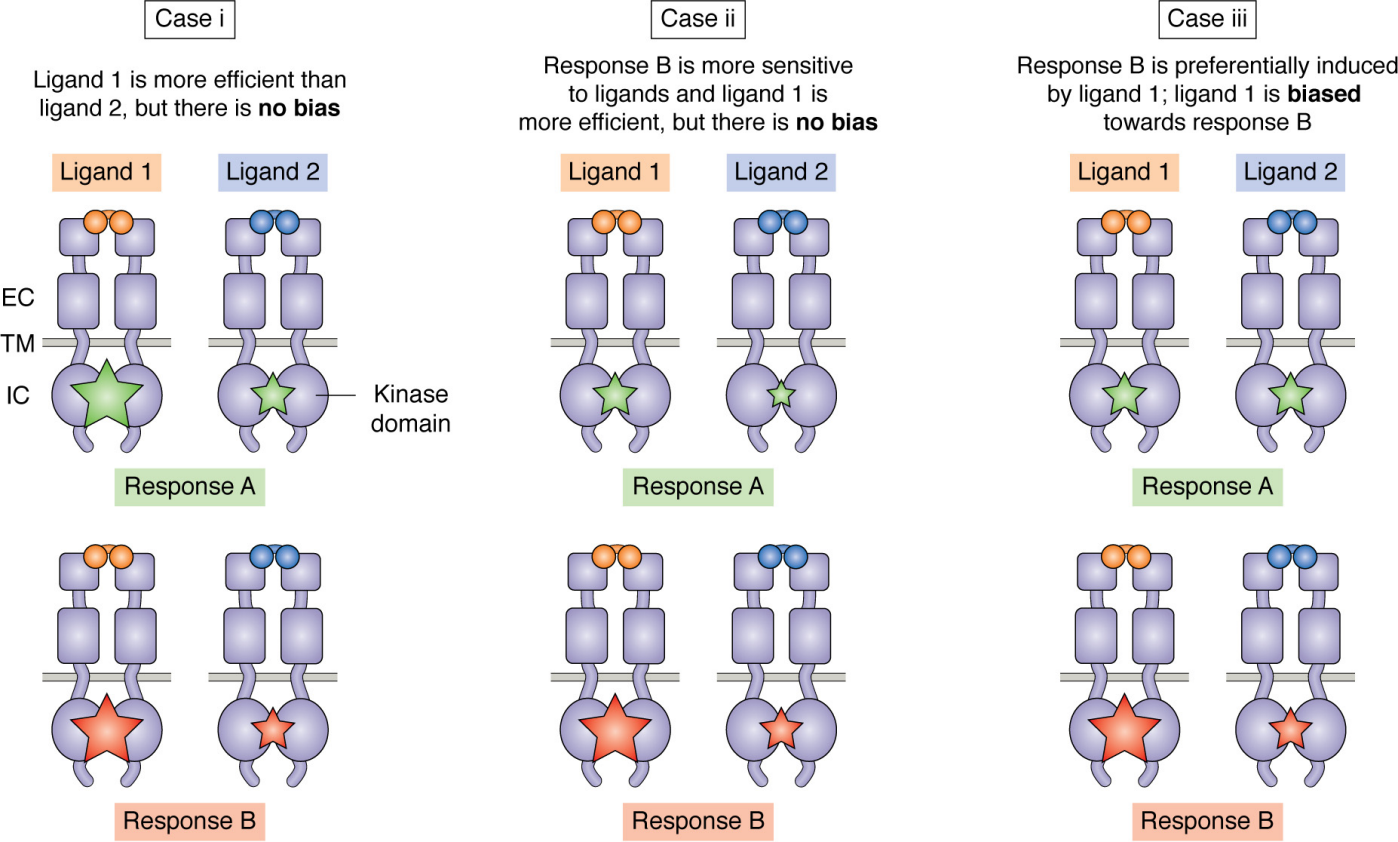
**Ligand bias can lead to fundamentally different receptor signaling outcomes.** A schematic illustrates different cases in which two ligands induce dimerization of a RTK and downstream signaling. The *stars* represent two signaling responses: response A in *green* and response B in *red*. The *size* of the *stars* represents the efficiency of a response (*i.e.* a combination of the potency of the ligand and the magnitude (or efficacy) of the response) (Table 1). In **case i**, ligand 1 induces both responses more efficiently than ligand 2, but the two responses are equally increased with ligand 1, and therefore there is no bias. In **case ii**, response B is more efficient for both ligands, and in addition ligand 1 induces both responses more efficiently, but there is no bias in the responses induced by the two ligands. There is bias only in **case iii**, where only response B is more efficiently induced by ligand 1 compared with ligand 2, and thus ligand 1 is biased toward response B. *EC*, extracellular region; *TM*, transmembrane helix; *IC*, intracellular region.

There have been reports that propose the existence of ligand bias for various RTK families. These include the ERBB receptor family (7, 21, 22, 23, 24, 25), the fibroblast growth factor receptor (FGFR) family (26, 27), the TRK receptor family (28, 29, 30, 31, 32, 33), the insulin receptor family (34, 35, 36, 37, 38, 39, 40, 41, 42), the platelet-derived growth factor receptor (PDGFR) family (43), the RET receptor (44), and the EPH receptor family (45, 46). It is conceivable that many, if not all, RTKs engage in biased signaling (9), but this aspect of their signaling activity has not been thoroughly investigated thus far. To accelerate progress in the study of RTK signaling bias, we can use existing tools developed for GPCRs.

A goal of this review is to highlight the concept of ligand bias in RTK signaling and to outline protocols that can be used to identify and quantify bias by analyzing dose-response curves. A second goal is to provide insights into the biophysical basis of RTK ligand bias and give an overview of mechanistic hypotheses proposed in the literature to explain ligand bias. A third goal is to encourage new research into RTK ligand bias and the mechanisms that underpin it.

## Fundamentals of ligand bias

### Quantitative differences in signaling responses and ligand bias

“Ligand bias” or “biased agonism” is the ability of distinct ligands to differentially activate specific signaling responses downstream of a single receptor. A “response” is defined as an effect due to ligand binding and may include receptor phosphorylation and changes in components of downstream signaling pathways. Responses could also include effects on cell behavior that depend on complex coordination of multiple signaling pathways, such as changes in cell proliferation, metabolism, differentiation, or migration. Ligand bias can lead to fundamental differences in the signaling output of a RTK in response to different ligands (2, 5). It does not just reflect, for example, similar quantitative differences in all of the signaling responses induced by different ligands.

This is illustrated in Fig. 1 for two ligands that activate the same two signaling responses, A and B, through the same RTK. **Cases i** and **ii** show examples of quantitative differences in the responses induced by the two ligands, but without bias. In **case i**, ligand 1 induces more efficient responses than ligand 2, but the two responses are equally increased with ligand 1 and therefore are not biased. Ligand 1 is also more efficient in **case ii**, and in addition, response B is more efficient than response A for both ligands. However, the variation in the two responses is the same for both ligands, and therefore the quantitative differences shown in **case ii** also do not represent ligand bias. **Case iii** illustrates an example of ligand bias. In **case iii**, both ligands activate response A similarly, but ligand 1 activates response B more efficiently than ligand 2. Only in this case the two responses are differentially activated by the two ligands, and ligand 1 is biased toward response B.

The identification and quantification of bias is not trivial. Ligand bias is a property of the ligand/receptor system and its coupling to downstream signaling responses. To determine whether signaling is biased or not, at least two ligands and at least two responses need to be evaluated, as illustrated in Fig. 1. Furthermore, dose-response curves have to be acquired because parameters obtained from fitting such curves are needed for the quantification of ligand bias (1, 47, 48) (see below). In addition, as emphasized in the GPCR literature, to arrive at a correct assessment of signaling bias, it is important to eliminate potential bias due to the experimental system (such as, for example, the cellular context) and the type of measurement or assay used (1, 2, 5, 6, 48). Correctly quantified bias should not be influenced by these factors.

Properly designed experiments can involve measuring different responses in different cell lines and with different assay systems. However, a response induced by different ligands should be measured using the same assay system and in the same cell line. Thus, experimental designs that should be avoided include measuring (i) the response to a ligand in one cell line and the same response to a different ligand in a different cell line or (ii) the response to a ligand with one assay and the same response to a different ligand with a different assay. It is also important to acquire a complete dose-response curve over a broad ligand concentration range rather than using only one or several ligand concentrations.

### Identifying and quantifying ligand bias: A practical demonstration

This section illustrates how bias can be quantified for RTKs using protocols borrowed from the GPCR field. The data we use for this demonstration have been published previously (49). We chose two engineered peptide ligands that activate the EphA2 receptor that was endogenously expressed in the PC3 prostate cancer cell line. The two peptides, designated here YSK (βAWLAYPDSVPYSK-biotin) and YSPK (βAWLAY-PDSVPYSPK-biotin), differ only by one residue (Pro-13 in YSPK, which is not present in YSK). Both peptides induce two responses that are also induced by the natural ephrinA ligands (49). Response A involves phosphorylation of Tyr-588 in the juxtamembrane region of EphA2, which is an autophosphorylation site known to promote receptor kinase activity. Response B involves inhibition of AKT phosphorylation on Ser-473. EphA2 Tyr-588 phosphorylation and AKT Ser-473 phosphorylation were detected in cells treated for 15 min with different peptide concentrations by immunoblotting cell lysates using phosphospecific antibodies. The phosphorylation signals were quantified by measuring chemiluminescence in the immunoblots.

**Figure 2:**
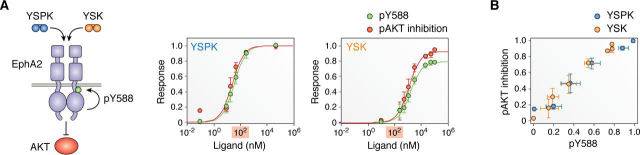
**Dose-response curves and bias plots for ligand bias assessment.***A*, schematic illustrating the two ligand-induced EphA2 signaling responses analyzed and dose-response curves for two peptide ligands, YSPK and YSK, that activate the EphA2 RTK in PC3 prostate cancer cells. The two responses measured are autophosphorylation on tyrosine 588 (*pY588*, which is normalized to total EphA2 levels) and inhibition of AKT phosphorylation (*pAKT*). The 100 nm concentration is *highlighted* in the graphs to emphasize the different potency of the two ligands. *B*, bias plot comparing the two responses for the two ligands. The *color coding* for the two responses (*green* and *red*) and the two ligands (*blue* and *orange*) are the same as in Fig. 1.

In the original publication (49), dose-response curves were scaled individually for each peptide because the data were not used to investigate ligand bias. Here, we rescaled the data by normalizing the data for both peptides to the maximal response observed. This is the response induced by saturating concentrations of the YSPK peptide, which was chosen as the reference ligand. Fig. 2*A* shows EphA2 Tyr-588 phosphorylation and pAKT inhibition (*i.e.* inhibition of AKT Ser-473 phosphorylation) as a function of ligand concentration for the two peptide ligands. The data for each curve are from at least four independent experiments.

When comparing the dose-response curves for the two ligands in Fig. 2*A*, we see that a lower concentration of YSPK is needed to reach the maximum EphA2 phospho-Tyr-588 (*pY588*) phosphorylation level, and therefore the YSPK peptide is much more potent than the YSK peptide. Much lower YSPK concentrations stimulate both EphA2 Tyr-588 phosphorylation (EC_50_ values are ∼40 nm for YSPK and ∼1,400 nm for YSK) and AKT inhibition (EC_50_ values are ∼30 nm for YSPK and ∼1,000 nm for YSK). The maximal responses (*E*_top_) are also different. However, this does not necessarily mean that the YSK ligand is biased compared with the YSPK reference ligand (see Fig. 1, **case ii**). To determine whether significant ligand bias exists or not, we can create a bias plot to visualize potential bias. Furthermore, we need to calculate a bias coefficient that takes into account the relationship of EC_50_ and *E*_top_ for different ligands and responses.

The bias plot (Fig. 2*B*) is a graphical representation that shows response A (Tyr-588 phosphorylation) as a function of response B (pAKT inhibition) for each ligand (2). The bias plot is directly derived from the dose-response data, by plotting the magnitude of response A *versus* the magnitude of response B for each ligand concentration. The bias plot reports on the relative ability of a ligand to induce the two responses, without the need for assumptions or mathematical modeling (2). The data points for different ligands can be directly compared because they incorporate system and measurement bias in identical ways. We see that the YSK data points in Fig. 2*B* overlap with the YSPK data points, despite the fact that one ligand is more potent than the other. This suggests that there is no bias in the responses induced by the two ligands. Note that this analysis can be used even if response A for the two ligands is measured in one cell line and response B in a different cell line. As long as each response is measured in the same way for both ligands (2, 3, 50), differences between the two ligands in the bias plot are entirely due to ligand bias.

Quantification of bias involves the calculation of a single numerical value (a bias coefficient) for each ligand, which reports on the existence and magnitude of ligand bias (48, 51). Different types of bias coefficients are described in the GPCR literature, and readers are encouraged to familiarize themselves with them (2, 3, 5, 51, 52, 53). Here, we demonstrate how to calculate a bias coefficient known as “β_lig_” (48). β_lig_ was chosen from several available mathematical formalisms to assess bias, because it can yield an accurate description of GPCR bias (53) and because it is based on simple mathematical equations. In the next section, we confirm that β_lig_ can be used to evaluate bias for RTKs.

Determination of β_lig_ starts by fitting each dose-response data set according to a phenomenological equation describing a simple binding curve (sometimes referred to as “binding isotherm” or “Langmuir isotherm,” corresponding to a “Hill equation” with Hill coefficient *n* = 1),

(Eq. 1) $$Response=\frac{\left[L\right]E_{top}}{[L]+{EC}_{50}}$$

where [L] is the free ligand concentration, assumed to be similar to the total concentration, *E*_top_ is the maximal response observed in the dose-response curve (the plateau at high ligand concentration), and EC_50_ is the ligand concentration that induces a response corresponding to 50% of *E*_top_ (Table 1). The fitted curves are shown as *solid lines* in Fig. 2*A*. Curve fitting yields the values of EC_50_ and *E*_top_, which are then used to calculate the bias coefficient β_lig_ (48).

**Table 1:** Definition of parameters

Parameter	Definition
*E*_top_	Maximal response in the Hill equation; efficacy
EC_50_	Ligand concentration that induces a response corresponding to 50% of *E*_top_; potency
*E*_max_	Maximal response that can be induced by any ligand
*K*_resp_	Concentration of ligand-bound receptors that produces 50% of *E*_max_. *K*_resp_ is a measure of the efficiency of the response; when *K*_resp_ is low, efficiency is high
τ	Transduction coefficient
*K*_L_, $K_{L}^{\prime }$, $K_{L}^{\prime \prime }$	Ligand-receptor dissociation constants
*K*_R_, $K_{R}^{\prime }$, $K_{R}^{\prime \prime }$	Receptor-receptor dissociation constants

The ratio of the two fitted parameters, *E*_top_/EC_50_, has been recognized as an important descriptor of ligand efficiency (54), and ligand bias coefficients can be calculated using these ratios for different ligands and responses. In particular, the equation to calculate β_lig_ is as follows (48, 54),

(Eq. 2) $$\beta_{lig}=log\left({\left(\frac{{E_{top,A}EC}_{50,B}}{{EC}_{50,A}E_{top,B}}\right)}_{lig}{\left(\frac{{E_{top,B}EC}_{50,A}}{{EC}_{50,B}E_{top,A}}\right)}_{ref}\right)$$

where “lig” indicates the ligand (YSK) being compared with the reference ligand “ref” (YSPK), and “A” and “B” are the two responses, Tyr-588 phosphorylation and pAKT inhibition. Thus, based on this equation, the value of β_lig_ for the reference ligand (in our case, YSPK) is 0 (*i.e.* β_YSPK_ = 0). Using Equation 2, we calculated β_YSK_ as −0.02 ± 0.12. This value is not significantly different from β_YSPK_ = 0, based on a one-sample *t* test. Thus, YSK is an unbiased ligand when compared with YSPK, despite being less potent.

Equation 2 can be applied to other published data. For instance, we used it to compare the c-Kit ligand stem cell factor and an engineered partial agonist derivative with impaired ability to promote dimerization of the c-Kit receptor (43). We used the dose-response curves for these two ligands as reported and fitted by Ho *et al.* (43) in Fig. 5 (*B* and *D*) of their article. In these dose-response curves, they analyzed *ex vivo* proliferation of hematopoietic stem and progenitor cells purified from mouse bone marrow (response A) and the release of IL-6 by mouse bone marrow–derived cultured mast cells (response B). Using Equation 2, we calculated the value of β_lig_ for the engineered ligand as compared with WT stem cell factor as the reference ligand. This β_lig_ value (−0.72 ± 0.16) is significantly different from 0, supporting the conclusion that the engineered ligand is biased toward cell proliferation and against IL-6 secretion (43).

This approach can be used to determine whether bias exists or not, provided that the data are well-described by Equation 1. In our experience, Equation 1 generally gives a reasonable fit to RTK dose-response curves. If it is necessary to use a Hill equation with *n* ≠ 1 to fit the data, then a more complex method needs to be used to calculate bias (2, 3, 53). However, it has been suggested that Equation 2 can be used even if *n* ≠ 1, as long as *E*_top_ > 0.35 and *n* > 0.5 (54).

## Mathematical models of receptor activation: insights into ligand bias

### GPCRs

In the previous section, we demonstrated that Equation 1 can be used to fit experimental dose-response curves. Whereas the approach we used is phenomenological (*i.e.* Equation 1 is based on the shape of experimental dose-response curves), there are mechanistic models of receptor activation. In this section, we outline a physicochemical mechanistic model of GPCR activation and show how the parameters in the phenomenological Equation 1 relate to the parameters of this mechanistic model.

Many physicochemical models of GPCR activation that vary in sophistication have been developed (55, 56, 57). The simplest, and perhaps most widely used, is the so-called “operational model” introduced by Black and Leff (58). This model is based on the experimental observation that the relationship between the concentration of activated receptors and a measured response is often hyperbolic. In other words, this model assumes that the ligand-bound receptor, RL, activates the cellular response with an effective equilibrium dissociation constant denoted as *K*_resp_ according to the equation,

(Eq. 3) $$Response=\frac{\left[RL\right]E_{max}}{[RL]+K_{resp}}$$

Mathematically, *K*_resp_ is the concentration of ligand-bound receptors that produces 50% of the largest possible response that can be achieved by the system (*E*_max_) (Table 1). Data are often normalized so that *E*_max_ = 1. Physically, *K*_resp_ describes the propensity of the ligand-bound receptor to induce a response. Thus, *K*_resp_ is a measure of the efficiency of the response: the more efficient the process, the smaller the value of *K*_resp_ (Table 1).

The concentration of the ligand-bound receptor [RL] in Equation 3 depends on the concentrations of free receptor [R] and ligand [L] and on the ligand-receptor dissociation constant *K*_L_ according to the following equation.

(Eq. 4) $$\left[RL\right]=\frac{\left[R\right]\left[L\right]}{K_{L}}$$

The total receptor concentration [Rt] is as follows.

(Eq. 5) $$\left[Rt\right]=\left[R\right]+\left[RL\right]$$

Therefore, substitution of Equation 5 into Equation 4 yields the following.

(Eq. 6) $$\left[RL\right]=\frac{\frac{\left[Rt\right]\left[L\right]}{K_{L}}}{1+\frac{\left[L\right]}{K_{L}}}$$

Substitution of Equation 6 into Equation 3 yields the following.

(Eq. 7) $$Response=\frac{\left[Rt\right]\left[L\right]E_{max}}{\left[Rt\right][L]+{K_{resp}(K_{L}+\left[L\right])}_{}}=\frac{\left[Rt\right]\left[L\right]E_{max}}{\left[L\right](\left[Rt\right]+{K_{resp})+(K_{L}K_{resp})}_{}}$$

If we divide both the numerator and the denominator by *K*_resp_, we obtain Equation 8,

(Eq. 8) $$Response=\frac{\left(\frac{\left[Rt\right]}{K_{resp}}\right)\left[L\right]E_{max}}{\left(\frac{\left[Rt\right]}{K_{resp}}+1\right){\left[L\right]+(K_{L})}_{}}=\frac{\text{τ}\left[L\right]E_{max}}{(\text{τ}+1){\left[L\right]+(K_{L})}_{}}$$

Equation 8 is the “operational model,” and τ is the “transducer coefficient” defined as follows.

(Eq. 9) $$\text{τ}=\frac{\left[Rt\right]}{K_{resp}}$$

Equation 8 can also be written as follows.

(Eq. 10) $$Response=\frac{\tau /(\tau +1)\left[L\right]E_{max}}{\left[L\right]{+(K_{L})/(\tau +1)}_{}}$$

Equation 10 is the same as Equation 1, where the following is true.

(Eq. 11) $$E_{top}=\frac{\tau E_{max}}{\left(\tau +1\right)}$$

(Eq. 12) $${EC}_{50}=\frac{K_{L}}{\left(\tau +1\right)}$$

Thus, the Black and Leff model provides a justification for using the simple phenomenological Equation 1 when fitting dose-response curves for GPCRs. Additional models have been developed in the GPCR field, including the “ternary complex” (55), “extended ternary complex” (56), and “cubic ternary complex” (57, 59) models. These models incorporate specific interactions of the GPCR with signaling proteins that mediate the downstream responses. Within the operational model, the effects of these interactions are incorporated into the *K*_resp_ value.

We can now ask which parameters of the Black and Leff model carry information about ligand bias. To answer this question, we substitute the expressions in Equations 11 and 12 into Equation 2 and simplify to obtain the following.

(Eq. 13) $$\beta_{lig}=\log\left({\left(\frac{{E_{top,A}EC}_{50,B}}{{EC}_{50,A}E_{top,B}}\right)}_{lig}{\left(\frac{{E_{top,B}EC}_{50,A}}{{EC}_{50,B}E_{top,A}}\right)}_{ref}\right)=log\left({\left(\frac{{{\text{τ}}_{A}K}_{L,B}}{K_{L,A}{\text{τ}}_{B}}\right)}_{lig}{\left(\frac{{{\text{τ}}_{B}K}_{L,A}}{K_{L,B}{\text{τ}}_{A}}\right)}_{ref}\right)$$

This equation shows that β_lig_ depends on the values of the ligand-receptor dissociation constant *K*_L_ and the transducer coefficient τ for both the ligand of interest and the reference ligand. Whereas it is known that ligand-binding affinity can be influenced by the identity of the downstream molecules that interact with the receptor (3), here we assume that *K*_L_ does not depend on the particular receptor signaling response measured in the dose-response curves. This will be a valid assumption when the measured responses are averaged for receptors bound to multiple downstream signaling proteins, as is the case for many types of receptors in the cellular environment.

Thus, *K*_L_ for response A (*K*_L,A_) is the same as *K*_L_ for response B (*K*_L,B_), and Equation 13 can be simplified as follows.

(Eq. 14) $$\beta_{lig}=\log\left({\left(\frac{{\text{τ}}_{A}}{{\text{τ}}_{B}}\right)}_{lig}{\left(\frac{{\text{τ}}_{B}}{{\text{τ}}_{A}}\right)}_{ref}\right)=log\left({\left(\frac{{\left[R_{t,A}\right]/K}_{resp,A}}{{\left[R_{t,B}\right]/K}_{resp,B}}\right)}_{lig}{\left(\frac{{\left[R_{t,B}\right]/K}_{resp,B}}{{\left[R_{t,A}\right]/K}_{resp,A}}\right)}_{ref}\right)$$

The concentration of the receptors [Rt] is also independent of the type of response measured in the experiments (*i.e.* $\left[R_{t,A}\right]=\left[R_{t,B}\right]$), and thus the following is true.

(Eq. 15) $$\beta_{lig}=\log\left({\left(\frac{K_{resp,B}}{K_{resp,A}}\right)}_{lig}{\left(\frac{K_{resp,A}}{K_{resp,B}}\right)}_{ref}\right)=log\left({\frac{{{(K}_{resp,A})}_{ref}}{{{(K}_{resp,A})}_{lig}}}_{}\frac{{{(K}_{resp,B})}_{lig}}{{{(K}_{resp,B})}_{ref}}\right)$$

Equation 15 shows that bias arises due to differences in *K*_resp_, the parameter describing the coupling of the ligand-bound receptor to downstream signaling responses. It allows us to formulate mathematically the conditions resulting in bias. There is bias when the argument of the logarithm is significantly different from 1 (and β_lig_ is significantly different from 0). In other words:

(Eq. 16) $$\frac{{{(K}_{resp,A})}_{lig}}{{{(K}_{resp,A})}_{ref}}\neq \frac{{{(K}_{resp,B})}_{lig}}{{{(K}_{resp,B})}_{ref}}bias$$

and

(Eq. 17) $$\frac{{{(K}_{resp,A})}_{lig}}{{{(K}_{resp,A})}_{ref}}=\frac{{{(K}_{resp,B})}_{lig}}{{{(K}_{resp,B})}_{ref}}nobias$$

Equation 15 shows that the ligand-receptor dissociation constant *K*_L_ has no relevance for ligand bias. It also shows that the value of β_lig_ is related to *K*_resp_, in agreement with the definition of ligand bias. This provides a justification for the utility of Equation 2 in bias quantification and clarifies the physical meaning of β_lig_. In practice, β_lig_ is calculated using Equation 2 and then analyzed for significance as illustrated in the previous section. Generally, *K*_resp_ cannot be calculated from Equation 3 because the concentration [RL] is unknown in most experiments.

### RTKs

In this section, we show that a mathematical formalism that is analogous to the GPCR operational model can be developed for RTKs to help us understand RTK ligand bias. The operational model of Black and Leff describes the case of one ligand binding to one GPCR. On the other hand, RTK activation generally requires receptor dimerization and sometimes oligomerization. To develop an operational model of RTK activation, we will consider a model in which ligand binding is coupled to RTK dimerization. Different models should be used for RTKs that are constitutively dimerized through disulfide bonds (which can be described using Equation 4) and for RTKs that form oligomers larger than dimers (which require more complicated equations) (60, 61). This coupling can be understood though the use of thermodynamic cycles, which show how increases in both receptor and ligand concentration drive the transition to active liganded RTK dimers via intermediate states.

**Figure 3:**
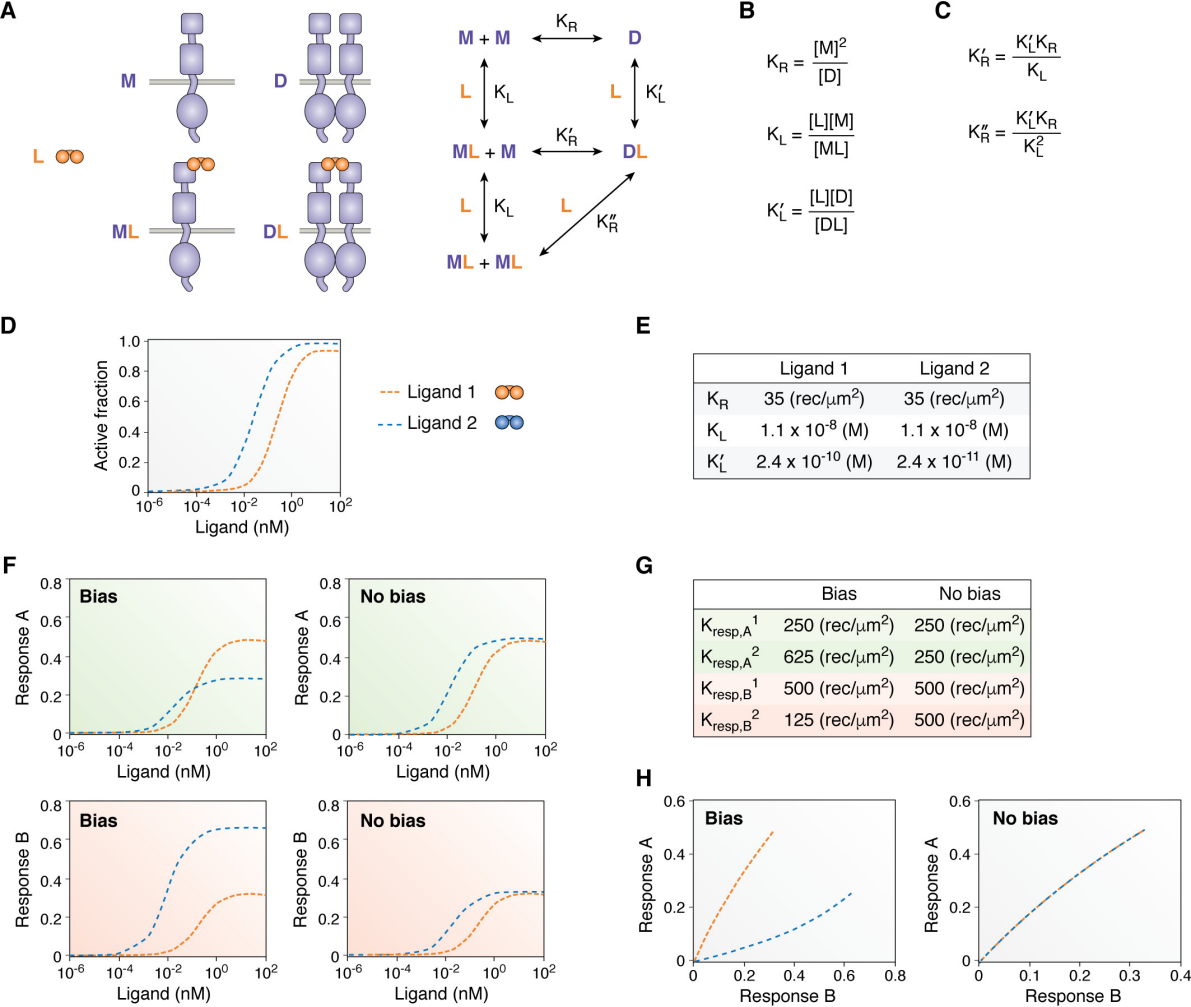
**Predictions of responses for dimeric ligands based on the RTK operational model.***A*, schematic and thermodynamic cycle describing VEGFA binding to VEGFR2 and VEGFR2 dimerization. *B*, definitions of the three principal dissociation constants. *C*, definition of the other dissociation constants in terms of the three principal ones. *D*, predicted *orange curve*: abundance of VEGFR2 dimers bound to VEGFA [DL] as a function of VEGFA (ligand 1) concentration. Predicted *blue curve*, abundance of VEGFR2 dimers [DL] bound to a hypothetical ligand 2 as a function of ligand 2 concentration. *E*, measured dissociation constant values for VEGFA from Ref. 60 and assigned dissociation constant values for the hypothetical ligand 2, which binds with 10-fold higher affinity than ligand 1 to the VEGFR2 dimer. *F*, dose-response curves predicted using Equation 24 based on the values shown in *G*. *G*, values of *K*_resp_ for responses A or B and for ligands 1 or 2 that were used to simulate the cases of bias and no bias in *F*. In all cases, [Rt] = 500 receptors/µm^2^. *H*, bias plots calculated from the predictions in *F*.

Fig. 3*A* describes the simplest mode of RTK activation, which involves the binding of a dimeric ligand (such as a constitutive dimer of two identical polypeptides) to its receptor (as shown in Fig. 3*A*). This activation mode is used, for example, by vascular endothelial growth factor A (VEGFA) to activate VEGF receptor 2 (VEGFR2) in a process that is critical for angiogenesis (62, 63). The ligand dissociation constants in the cycles are denoted as *K*_L_ and $K_{L}^{\prime }$, whereas the dissociation constants describing receptor-receptor lateral interactions are denoted as *K*_R_, $K_{R}^{\prime }$, and $K_{R}^{\prime \prime }$. Three of these equilibrium dissociation constants are sufficient to define this model: *K*_L_, the ligand-receptor dissociation constant for monomeric VEGFR2; $K_{L}^{\prime }$, the ligand-receptor dissociation constant for dimeric VEGFR2; and K_R_, the receptor-receptor dissociation constant for VEGFR2 in the absence of ligand. The definitions of these three constants are given in Fig. 3*B*. The values of *K*_L_, $K_{L}^{\prime }$, and *K*_R_ for VEGFA binding to VEGFR2 are reported in the literature (60) (Fig. 3*E*, ligand 1), and the other dissociation constants in the cycle can be determined from them, as shown in Fig. 3*C*. The knowledge of *K*_L_, $K_{L}^{\prime }$, and *K*_R_ allows determination of the ligand occupancy of the different monomeric and dimeric states of the receptor for any total receptor and ligand concentrations. Of particular interest is the concentration of the VEGFA-bound VEGFR2 dimer [DL], which accounts for all of the fully activated receptor (Fig. 3*D*, *orange dotted line*). Ligand binding causes a conformational change that is required to activate the receptor dimer (64, 65), and therefore, the unliganded VEGFR2 dimer has very low activity. In addition, unliganded and liganded VEGFR monomers are completely inactive.

To develop an operational model that links ligand-receptor interactions that occur extracellularly to intracellular responses, we follow the formalism of Black and Leff and use Equation 3 but substitute the concentration of ligand-bound receptor [RL] with the concentration of ligand-bound dimer [DL] to obtain Equation 18,

(Eq. 18) $$Response=\frac{[DL]E_{max}}{[DL]+K_{resp}}$$

*K*_resp_ in Equation 18 is the concentration of liganded dimer [DL] that produces 50% of the maximum possible response. For RTKs, *K*_resp_ can be considered as an equilibrium constant that describes the efficiency of the response of interest, including autophosphorylation, interaction with a binding partner, activation of a downstream signaling molecule, or changes in cellular behavior. As with GPCRs, the more efficient the process, the smaller the value of *K*_resp_. *E*_max_ is the largest possible response that can be observed for the system with any ligand and will be set to 1.

The concentration of active dimer [DL] is given by Equation 19,

(Eq. 19) $$\left[DL\right]=\frac{{\left[M\right]}^{2}[L]}{K_{L}^{\prime }K_{R}}$$

where $K_{L}^{\prime }$ and *K*_R_ are the dissociation constants defined in Fig. 3 (*A* and *B*), [M] is the concentration of receptor monomers, and [L] is the free ligand concentration. [M] is unknown, but it can be determined if the total receptor concentration [Rt] is known.

(Eq. 20) $$\left[Rt\right]=\left[ML\right]+\left[M\right]+2\left[D\right]+2\left[DL\right]$$

Substituting the dissociation constants defined in Fig. 3 (*A* and *B*) for [ML], [D], and [DL] in Equation 20 yields a quadratic equation in terms of [M].

(Eq. 21) $$[Rt]=\frac{\left[M\right]\left[L\right]}{K_{L}}+\left[M\right]+\frac{{2\left[M\right]}^{2}}{K_{R}}+\frac{2{\left[M\right]}^{2}\left[L\right]}{K_{L}^{\prime }K_{R}}$$

Accordingly, [M] can be solved for in terms of [L], [Rt], and the dissociation constants.

(Eq. 22) $$\left[M\right]=\frac{\frac{-(\left[L\right]+K_{L})}{K_{L}}+\sqrt{{\left(\frac{\left[L\right]+K_{L}}{K_{L}}\right)}^{2}+8\left(\frac{K_{L}^{\prime }+[L]}{K_{L}^{\prime }K_{R}}\right)[Rt]}}{4\left(\frac{K_{L}^{\prime }+[L]}{K_{L}^{\prime }K_{R}}\right)}$$

Equation 22 can then be substituted into Equation 19 as follows.

(Eq. 23) $$\left[DL\right]=\frac{{\left(\frac{\frac{-\left(\left[L\right]+K_{L}\right)}{K_{L}}+\sqrt{{\left(\frac{\left[L\right]+K_{L}}{K_{L}}\right)}^{2}+8\left(\frac{K_{L}^{\prime }+[L]}{K_{L}^{\prime }K_{R}}\right)[Rt]}}{4\left(\frac{K_{L}^{\prime }+[L]}{K_{L}^{\prime }K_{R}}\right)}\right)}^{2}[L]}{K_{L}^{\prime }K_{R}}$$

Finally, Equation 23 can be substituted into Equation 18 to express the response as a function of the dissociation constants that govern RTK ligand binding and dimerization as follows.

(Eq. 24) $$Response=\frac{\frac{{\left(\frac{\frac{-\left(\left[L\right]+K_{L}\right)}{K_{L}}+\sqrt{{\left(\frac{\left[L\right]+K_{L}}{K_{L}}\right)}^{2}+8\left(\frac{K_{L}^{\prime }+[L]}{K_{L}^{\prime }K_{R}}\right)[Rt]}}{4\left(\frac{K_{L}^{\prime }+[L]}{K_{L}^{\prime }K_{R}}\right)}\right)}^{2}}{K_{L}^{\prime }K_{R}}E_{max}[L]}{\frac{{\left(\frac{\frac{-\left(\left[L\right]+K_{L}\right)}{K_{L}}+\sqrt{{\left(\frac{\left[L\right]+K_{L}}{K_{L}}\right)}^{2}+8\left(\frac{K_{L}^{\prime }+[L]}{K_{L}^{\prime }K_{R}}\right)[Rt]}}{4\left(\frac{K_{L}^{\prime }+[L]}{K_{L}^{\prime }K_{R}}\right)}\right)}^{2}[L]}{K_{L}^{\prime }K_{R}}+K_{resp}}$$

Equation 24 for the binding of a dimeric ligand to a RTK is the analog of Equation 7 for GPCRs but is more complicated due to the coupling between ligand binding and receptor dimerization. In the case of GPCRs, we simplified Equation 7 to yield Equation 10, which is the same as Equation 1. However, we cannot similarly simplify the more complicated Equation 24. Nevertheless, Equation 24 allows us to simulate the response for any value of the equilibrium constants shown in Fig. 3*A*. In Fig. 3*F*, we show examples of simulated responses for different VEGFA ligand concentrations (*orange dotted lines*) using the experimentally measured values of *K*_L_, $K_{L}^{\prime }$, and *K*_R_ (Fig. 3*E*) and hypothetical values of *K*_resp_ for responses A and B (Fig. 3*G*). Thus, we can use Equation 24 to simulate dose-response curves and determine whether Equation 1 can be used to fit these simulated dose-response curves in a meaningful way.

In a previous section, we used Equation 1 to fit experimental dose-response curves obtained for the EphA2 RTK activated by two engineered peptide ligands and Equation 2 to calculate bias coefficients and draw conclusions about the existence of bias. EphA2 is activated by the peptide ligands through a different mechanism than VEGFR2 by its ligand VEGFA because each biotinylated peptide is a monomer that bivalently interacts with two EphA2 molecules to ultimately form a complex of two peptides bound to two receptors (49). To investigate whether Equations 1 and 2 are applicable for analysis of bias in RTKs with different dimerization mechanisms, we first simulated dose-response curves for the cases of bias and no bias for two VEGFR2 ligands (Fig. 3*F*) and then asked whether these two cases can be correctly identified. Ligand 1 is the natural ligand, VEGFA, which we denote as the reference ligand. Ligand 2 is a hypothetical ligand that binds to the VEGFR2 monomer with the same affinity as VEGFA but binds with 10-fold higher affinity to the VEGFR2 dimer (Fig. 3, *E* and *F*). To simulate bias, we used different relative values of *K*_resp_ for the two different ligands and the two different responses, according to Equation 16 (Fig. 3*G*, *Bias*). To simulate the condition of no bias, we used the same relative values of *K*_resp_ for the two different ligands and the two different responses, according to Equation 17 (Fig. 3*G*, *No bias*). Indeed, *K*_resp_ is the only parameter in the operational model that accounts for the coupling of the RTK with signaling responses. Equations 16 and 17 are intuitive and are consistent with Fig. 1. Thus, they can be expected to be valid for both GPCRs and RTKs.

From the predicted dose-response data points, we can construct bias plots for response A *versus* response B for the two ligands (Fig. 3*H*). As expected, the curves for the two ligands are very different in the case of bias but overlap in the case of no bias. This suggests that bias plots, as developed for GPCRs, are also applicable for RTKs despite the fact that the RTK mechanism of activation is more complex.

Next, we fit the dose-response curves in Fig. 3*F* with Equation 1, and we determined the best fit *E*_top_ and EC_50_ values. Although we could not mathematically transform the complex Equation 24 into Equation 1, the fits with Equation 1 are very good (*R*^2^ > 0.9999), suggesting that Equations 1 and 24 have a very similar functional dependence on [L]. We then used Equation 2 to calculate the bias coefficients. We obtained β_lig_ = −1.01 in the bias case and β_lig_ = −0.01 in the no bias case. Thus, the β_lig_ value in the case of no bias is, as expected, very close to 0. β_lig_ is different from zero in the bias case, also as expected, suggesting that Equation 2 can successfully differentiate between the cases of RTK bias and no bias. Therefore, equations 1 and 2 can be a valuable tool in RTK research, provided that dose-response curves for at least two responses are measured.

A wealth of knowledge already exists about RTK signaling responses, and many tools are available to study them. For example, the major RTK downstream signaling pathways have been delineated, and phosphospecific antibodies are commercially available to detect phosphorylation events in the RTKs and in downstream signaling proteins. Bias plots and bias coefficients will reveal whether some of the RTK tyrosines are preferentially phosphorylated in response to a specific ligand, as compared with other ligands. These approaches can further reveal whether some pathways—such as the ERK mitogen-activated protein kinase, phophoinositide-3-kinase, protein kinase C, and signal transducer and activator of transcription pathways (10, 11, 17, 66, 67, 68)—are differentially activated by different ligands. If complex functional responses (such as cell proliferation, migration, differentiation, and metabolic responses) are measured as a function of ligand concentration, correlations may be revealed between the bias in the phosphorylation of specific tyrosines in the RTK and the bias in the activation of particular downstream signaling proteins or responses. Such correlations may suggest functional links that can be further investigated. Comparisons of different ligands using such quantitative assessments will yield fundamental knowledge on the regulation of RTK signaling responses that is not currently available.

To compare multiple ligands, it is useful to rewrite Equation 2 in the form,

(Eq. 25) $$\beta_{lig}=\beta_{lig}^{\prime }-\beta_{ref}^{\prime }$$

where $\beta_{lig}^{\prime }$ and $\beta_{ref}^{\prime }$ are defined as follows.

(Eq. 26) $$\beta_{lig}^{\prime }=log{\left(\frac{E_{top,A}{EC}_{50,B}}{{EC}_{50,A}E_{top,B}}\right)}_{lig}$$

(Eq. 27) $$\beta_{ref}^{\prime }=log{\left(\frac{E_{top,A}{EC}_{50,B}}{{EC}_{50,A}E_{top,B}}\right)}_{ref}$$

Multiple β′ values can then be compared using one-way analysis of variance to determine whether there are significant differences in the bias exhibited by multiple different ligands.

## Mechanisms of RTK ligand bias

In this section, we discuss molecular hypotheses that could explain how RTKs may differentially engage downstream signaling pathways depending on the bound ligand. For GPCRs, the explanation for this phenomenon could be that biased ligands stabilize distinctly different GPCR conformations that support preferential activation of a specific signaling pathway. For instance, the presence of multiple ligand-specific conformations of the β_2_-adrenergic receptor has been revealed using a quantitative MS approach (69). In addition, fluorine NMR experiments have shown that the cytoplasmic ends of helices VI and VII in the β_2_-adrenergic receptor adopt different conformations in response to G protein biased ligands and β-arrestin biased ligands (70). This view is supported by many other studies (5, 6, 71, 72, 73, 74) and is now the prevailing view in the field (75). Although dimerization can in some cases regulate GPCR signaling (75), it is the main mechanism of activation for RTKs. Thus, factors related to dimerization are particularly relevant for understanding RTK signaling bias.

### Do biased ligands stabilize different dimeric RTK catalytic conformations?

In analogy to the ligand-dependent conformational differences documented for GPCRs, it has been hypothesized that different ligands stabilize different RTK dimeric catalytic conformations, leading to ligand-specific preferential phosphor-ylation of selected tyrosines in the intracellular region (7). There is evidence that the transmembrane helix and the juxtamembrane segment in RTK dimers can sense the identity of the bound ligand and adopt different conformations (26, 65, 76). However, it is not known whether these conformational differences are transmitted to the kinase domain. Despite many years of research, it remains controversial whether the structural information about the bound ligand propagates to the kinase domain, such that different kinase-kinase interfaces are preferentially engaged in response to different ligands. Whereas some researchers believe that this is the case, others argue that linkers in RTKs (such as the linker connecting the extracellular region and the transmembrane helix or the juxtamembrane segment linker connecting the transmembrane helix and the kinase domain) are flexible. Thus, distinct structural changes that might occur in the extracellular region in response to the binding of different ligands may not be propagated to the intracellular region (24, 77, 78). If so, the two kinase domains in the dimer interact with each other based on their physicochemical properties and, therefore, in the same way, regardless of which ligand is bound to the extracellular region.

To resolve this controversy, it will be critical to monitor the configuration of the kinase domains to determine which kinase interfaces are used when an RTK is bound to different ligands. Researchers have long hoped that the structures of full-length RTKs would provide this information. The cryogenic EM (cryoEM) structures of the full-length insulin receptor bound to insulin and of the full-length insulin-like growth factor 1 receptor (IGF1R) bound to IGF1 have been recently reported (79, 80). In both cases, however, the intracellular regions are not resolved in the cryoEM maps. Only the extracellular regions are resolved, despite the fact that full-length receptors were analyzed. This likely indicates that the kinase domains explore many configurations within the active RTK dimer, consistent with the observation that tyrosines in different parts of the intracellular region undergo autophosphorylation. Because a substrate tyrosine must come in contact with the active-site cleft in the kinase domain to become phosphorylated, the kinase domains presumably exist in multiple configurations.

X-ray crystallography studies can characterize the structural arrangements of two dimerized kinase domains that are stabilized in RTK crystals. However, configurations that may be critically important for RTK autophosphorylation or for the binding and phosphorylation of downstream proteins may remain unresolved. Because there are many published high-resolution structures of RTK kinase domains, it may be useful to examine their crystallographic interfaces. It is possible that some of them are in fact biologically relevant interfaces that mediate kinase domain interactions within the context of the full-length RTK dimer in the plasma membrane. Indeed, analysis of crystal structures has identified interfaces that place a tyrosine in close proximity to the active site of the neighboring kinase (81, 82). A strategy to evaluate whether such contacts are important for biological function is to weaken the contacts through mutagenesis and then determine whether this perturbs the function of the full-length RTK (while not perturbing the folding of the kinase domain). If the mutations have effects on RTK signaling, they likely affect biologically relevant contacts. Such mutagenesis studies are rare (83) but can be very informative. There may also be interfaces that stabilize the kinase dimer but are not directly involved in phosphorylation and signaling. In this case, the interfaces can be identified by measuring RTK interactions rather than phosphorylation or functional outcomes.

If ligand bias arises from differential engagement of distinct interfaces that involve the kinase domain, the effects of destabilizing a particular interface will be different when different ligands are bound. Experiments to examine this hypothesis are feasible, because the effects of mutations in the putative kinase interfaces on dimer stability and phosphorylation can be measured using well-established techniques. These techniques include FRET (84, 85), co-immunoimmobilization (86), and fluorescence intensity fluctuations (87) to measure dimer stability as well as immunoblotting and mass spectrometry to measure tyrosine phosphorylation.

A possible outcome of these experiments is that a particular mutation will substantially affect RTK phosphorylation and/or dimer stability only when a specific ligand is bound but will have a much smaller effect when other ligands are bound. This would indicate that this specific ligand promotes a particular kinase domain interaction involving the mutated amino acid and that this interaction is not as important when other ligands are bound. This, in turn, would indicate that the identity of the bound ligand affects the arrangement of the two intracellular regions, supporting the view that structural information that depends on the identity of the ligand can be transmitted along the length of a RTK and reach the kinase domain.

Alternatively, it is possible that engineered mutations have the same effects on autophosphorylation and dimer stability, no matter what ligand is bound. This would suggest that kinase domain interactions and the pattern of phosphorylation sites do not depend on the identity of the bound ligand. This result would support the view that structural information related to the identity of the bound ligand does not reach the kinase domain. Such an outcome would suggest that factors other than the configuration of RTK molecules in a dimer or oligomer may differentially control signaling responses and ligand bias. A factor affecting bias that has been proposed in the literature is the stability of RTK dimers bound to different ligands, which is discussed in the next section.

### Can ligand bias be due to differences in the stability of RTK dimers bound to different ligands?

The stability of ligand-bound RTK dimers has been proposed to explain some instances of RTK ligand bias, because dimer stability can determine whether the response to a ligand is sustained or transient, which may ultimately affect biological outcomes (24, 27, 43, 88). In one example, the receptor-binding affinity of a ligand has been proposed to determine the strength and stability/durability of receptor dimerization to generate qualitatively different responses. This example involves the FGFR family, which plays a role in brain development (27, 89, 90). Two splice isoforms of the ligand FGF8 that differ by 11 amino acids (present at the N terminus of FGF8b but not FGF8a) have been shown to induce different phenotypes and gene expression patterns when electroporated in the midbrain of chicken embryos (89, 90). In particular, FGF8b induces the formation of the cerebellum by repressing the expression of the transcription factor orthodenticle homeobox 2 (Otx2). In contrast, FGF8a is not capable of repressing Otx2 and causes expansion of the optic tectum. Thus, these two FGF8 isoforms appear to act as biased ligands, because they induce different biological responses by activating the same receptor, FGFR1c (the c isoform of FGFR1, which is the major receptor for FGF8 in the developing midbrain). FGF8b binds with ∼10-fold higher affinity than FGF8a to the FGFR1c, FGFR2c, and FGFR3c extracellular domains. This effect is mediated by phenylalanine 32, a residue that is present only in FGF8b and contributes an additional hydrophobic contact with the receptor (89). Mutation of this phenylalanine to alanine weakens the FGFRc-binding affinity of FGF8b, functionally converting it into FGF8a. Thus, the binding affinity of the ligand for the receptor was proposed to be the defining factor that controls the functional outcome of receptor signaling. Furthermore, a connection was proposed between the stability of FGF8a-FGFRc or FGF8b-FGFRc dimers and the nature of the signaling, with the more stable FGF8b-FGFRc dimers transmitting stronger and more sustained intracellular signals (88).

Another study proposing that dimer stability correlates with ligand bias focuses on the epidermal growth factor receptor (EGFR), a member of the ERBB family that can be activated by multiple ligands (24). Some of them, such as EGF, bind with high affinity (apparent *K_d_* of 0.1–1 nm). Other ligands, such as epiregulin and epigen, bind 10–100 times more weakly. These ligands appear biased, as EGF has been shown to induce cell proliferation under conditions where epiregulin and epigen induce differentiation. The different cellular responses were found to correlate with the kinetics of EGFR activation. In the MCF-7 and T47D breast cancer cell lines, tyrosine phosphorylation of EGFR was sustained upon activation with epigen and epiregulin, whereas it was transient upon activation with EGF. The activation kinetics, in turn, were found to correlate with the stability of the different ligand-bound EGFR dimers. In particular, the stable EGF-bound EGFR dimers were proposed to induce a transient response, whereas the less stable epiregulin and epigen dimers were proposed to induce a more sustained response. The opposite effects of dimer stability on FGFR and EGFR activation kinetics are not well-understood but may depend on distinct mechanisms regulating the phosphorylation of the two RTK families and/or different interplays with phosphatases regulating their dephosphorylation (91, 92, 93).

**Figure 4:**
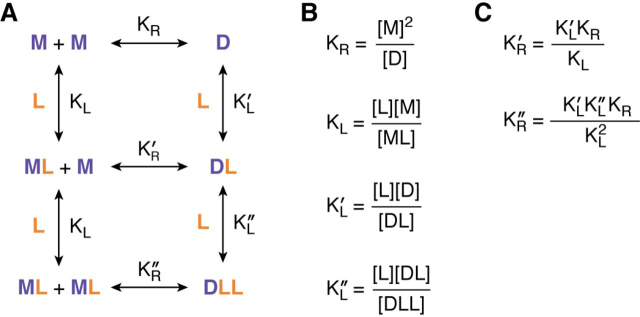
**Model describing the binding of a monomeric ligand to an RTK and RTK dimerization.***A*, thermodynamic cycle. *B*, definitions of the four principal equilibrium dissociation constants. *C*, definitions of the other equilibrium constants in terms of the four principal ones.

The hypothesis that RTK dimer stability is correlated with ligand bias has never been directly tested. Here, we outline an approach that can be used to examine the validity of this hypothesis. The term “dimer stability” is a thermodynamic parameter, also known as “dimerization free energy,” that reports on the strength of receptor dimerization and does not depend on receptor concentration. Accordingly, it can be defined using the receptor-receptor dissociation constants *K*_R_, $K_{R}^{\prime }$, and $K_{R}^{\prime \prime }$ (Figs. 3 (*A* and *B*) and 4).

EGF is a monomeric ligand that binds to a single receptor molecule, which is different from the case discussed above of dimeric VEGFA binding to VEGFR2. The thermodynamic cycle for a monomeric ligand (Fig. 4*A*) is more complex than the cycle for a dimeric ligand such as VEGFA (Fig. 3*A*) because the receptor dimers can bind either one or two monomeric ligands. As a consequence, there are three ligand-receptor dissociation constants denoted as *K*_L,_$K_{L}^{\prime }$, and $K_{L}^{\prime \prime }$ besides the three receptor-receptor dissociation constants denoted as *K*_R_, $K_{R}^{\prime }$, and $K_{R}^{\prime \prime }$. Four equilibrium dissociation constants are needed to define this cycle; their definitions are shown in Fig. 4*B*, and the links between the dissociation constants are shown in Fig. 4*C*.

*K*_R_ is the dissociation constant for dimerization of the unliganded monomeric receptor M, and the stability of the unliganded dimer is the dimerization free energy ΔG_R_ defined as follows,

(Eq. 28) $$\Delta G_{R}=RT\ln K_{R}$$

where *R* is the gas constant and *T* is the absolute temperature. Similarly, the dissociation constant $K_{R}^{\prime }$ describes the lateral interaction between an unliganded receptor monomer M and a liganded monomer ML, producing the dimer DL with a single ligand bound. The corresponding dimerization free energy is as follows.

(Eq. 29) $$\Delta G_{R}^{\prime }=RT{\ln K_{R}^{\prime }}^{}$$

Finally, the dissociation constant $K_{R}^{\prime \prime }$ describes the lateral interaction between two liganded receptor monomers ML, producing the dimer DLL with two ligands bound. The dimerization free energy associated with this process is as follows.

(Eq. 30) $$\Delta G_{R}^{\prime \prime }=RT{{\ln K_{R}^{\prime \prime }}^{}}^{}$$

The dimerization free energy values Δ*G*_R_, Δ$G_{R}^{\prime }$, and Δ$G_{R}^{\prime \prime }$ can be used to define the differences in the stability of receptor dimers with different ligand occupancy. For example, the extent of dimer stabilization due to the binding of two ligands is given by the following.

(Eq. 31) $$\Delta \Delta G_{lig}^{\prime \prime }=\Delta G_{R}^{\prime \prime }-\Delta G_{R}=RTln\frac{K_{R}^{\prime \prime }}{K_{R}}$$

This increase in stability can be quantified, because *K*_R_ and $K_{R}^{\prime \prime }$ can be measured in the plasma membrane of live cells using techniques such as FRET, co-immunoimmobilization, or fluorescence intensity fluctuations (84, 86, 87, 94). *K*_R_ can be measured in the absence of ligand, and $K_{R}^{\prime \prime }$ can be measured at saturating ligand concentrations. At saturating ligand concentrations, all receptors (both monomers and dimers) are ligand-bound, because the ligand concentration [L] is much higher than the ligand-receptor dissociation constants *K*_L_, $K_{L}^{\prime }$, and $K_{L}^{\prime \prime }$.

If a correlation exists between dimer stability and ligand bias, then the values of ΔΔ$G_{lig}^{\prime \prime }$ in Equation 31 and $\beta_{lig}^{\prime }$ in Equation 26 should be correlated. The correlation between ΔΔ$G_{lig}^{\prime \prime }$ and $\beta_{lig}^{\prime }$ measured for several ligands can be analyzed in a plot of $\beta_{lig}^{\prime }$ *versus* ΔΔ$G_{lig}^{\prime \prime }$, using either linear or nonlinear regression depending on the functional dependence.

It should be noted that within the context of the thermodynamic cycle–based RTK operational model, the information about bias depends entirely on the values of *K*_resp_, which have no direct link to dimer stability. Thus, we would expect no correlation between ΔΔ$G_{lig}^{\prime \prime }$ and $\beta_{lig}^{\prime }$ if the operational model provides a good description of RTK activation and RTK ligand bias. It is therefore important to probe this correlation experimentally, to directly verify whether a link exists between ligand bias and receptor dimer stability.

As discussed above, FGFR and EGFR dimer stability has been correlated with the kinetics of the response, either sustained or transient (24, 88). The factors affecting the kinetics of RTK autophosphorylation and dephosphorylation are complex and are not yet completely understood. For EGFR, it has been proposed that the kinetics of phosphorylation and dephosphorylation/degradation depend mainly on the identity of the ligand, but not on the ligand and receptor concentrations (24). However, other RTKs, such as EphA2, exhibit phosphorylation kinetics that depend strongly on ligand concentration (95). Currently, it is also not known how the kinetics of RTK autophosphorylation may contribute to ligand bias, as quantified using Equation 2. To gain insights, dose-response curves could be acquired at different time points, yielding time-resolved bias plots and time-resolved bias coefficients. This approach can reveal whether ligand bias depends on time for RTKs.

## Beyond biased ligands: Toward new biased modulators

It would be expected that not only activating ligands, but also other modulatory agents that bind to a receptor could bias downstream signaling responses by stabilizing a particular receptor structural conformation. For example, a characteristic of allosteric modulators is their ability to differentially affect distinct ligand-induced signaling responses, a phenomenon known as pathway bias (96, 97). Although this has been poorly studied so far, there are some examples of RTK inhibitors that act at allosteric sites and preferentially inhibit some of the responses induced by ligand binding, suggesting pathway-biased pharmacology (8). One of the examples is the small molecule AG1296, which targets the kinase domain of the PDGFRβ through a complex mechanism that depends on receptor activation state (98). AG1296 was shown to differentially inhibit PDGFR autophosphorylation on different tyrosines. Another example is the negative allosteric modulator SSR128129E, a small molecule that binds to the extracellular region of all FGFRs and induces a structural change that decreases fibroblast growth factor ligand efficacy (99, 100). SSR128129E was shown to inhibit tyrosine phosphorylation of an FGFR substrate (FRS2) but not another (PLCγ). Other interesting examples are matuzumab and cetuximab, two EGFR-inhibitory therapeutic antibodies with different binding sites in the EGFR extracellular region (101, 102). These antibodies can promote the formation of EGFR dimers that presumably have different configurations than the dimers induced by EGF but nevertheless can undergo autophosphorylation on at least some of the same tyrosines (102, 103). Despite inducing EGFR autophosphorylation, matuzumab and cetuximab do not activate the downstream AKT and ERK pathways (103). Similarly, artificial EGFR dimerization induced by bivalent synthetic ligands can induce autophosphorylation and recruitment of adaptor proteins, but not activation of AKT and ERK (104). Additional factors that could also bias signaling responses induced by ligands include receptor oligomerization, association with co-receptors, and subcellular trafficking (9). Thus, future studies will likely lead to a broader view of RTK biased signaling mechanisms and to the development of pathway-biased agents with novel modes of action.

## Prospective

Studies of GPCR biased signaling have revolutionized basic research and pharmacological discoveries (2, 6, 105, 106). It can be expected that an increased focus on RTK biased signaling will also transform the RTK field. It is enticing to think that studies of RTK ligand bias may reveal new biology that has been hidden from us due to a lack of quantification in our methodologies. Whereas the work is still in its early stages, it can progress rapidly if it takes advantage of the wealth of knowledge accumulated in the GPCR field and of the mathematical methods developed to identify biased agonism. Such rapid progress is highly desirable because the therapeutic implications of RTK ligand bias are far-reaching. We look forward to a new generation of smarter drugs that selectively target therapeutically relevant RTK signaling responses and have reduced side effects.

## Data availability

All data are included in the article.
